# Quantifying Change in Proteinuria After Acute Kidney Injury Among Patients With Chronic Kidney Disease: Findings From the Chronic Renal Insufficiency Cohort (CRIC) Study

**DOI:** 10.1016/j.xkme.2026.101272

**Published:** 2026-01-29

**Authors:** Y. Diana Kwong, Kathleen D. Liu, Alan S. Go, Anthony N. Muiru, Ian E. McCoy, Matthew R. Weir, Mark L. Unruh, Hernan Rincon-Choles, L. Lee Hamm, Jing Chen, Jesse Y. Hsu, Xiaoming Zhang, Chi-yuan Hsu, CRIC Study Investigators, Amanda H. Anderson, Lawrence J. Appel, Debbie L. Cohen, Laura M. Dember, James P. Lash, Mahboob Rahman, Panduranga S. Rao, Vallabh O. Shah

**Affiliations:** 1 Division of Nephrology, University of California San Francisco, San Francisco, CA; 2 Division of Research, Kaiser Permanente Northern California, Pleasanton, CA; 3 Division of Nephrology, Department of Medicine, University of Maryland Medical Center, Baltimore, MD; 4 Division of Nephrology, University of New Mexico, Albuquerque, NM; 5 Department of Kidney Medicine, Cleveland Clinic Foundation, Cleveland, OH; 6 Department of Medicine, Tulane University, New Orleans, LA; 7 Division of Nephrology, Charles and Jane Pak Center for Mineral Metabolism and Clinical Research, University of Texas Southwestern Medical Center, Dallas, TX; 8 Department of Epidemiology, Peter O'Donnell Jr School of Public Health, University of Texas Southwestern Medical Center, Dallas, TX; 9 Division of Biostatistics, University of Pennsylvania Perelman School of Medicine, Philadelphia, PA

**Keywords:** Acute kidney injury, proteinuria, cohort

## Abstract

**Rationale & Objective:**

Quantifying the change in proteinuria after acute kidney injury (AKI) may shed light on the pathway through which AKI contributes to kidney disease progression.

**Study Design:**

Prospective cohort study of patients with chronic kidney disease (CKD).

**Setting & Participants:**

3,197 participants who were enrolled in the multicenter, prospective Chronic Renal Insufficiency Cohort between January 7, 2013, and January 12, 2021.

**Exposure:**

AKI was defined as ≥1.5 peak to nadir inpatient serum creatinine ascertained during a hospital admission.

**Outcome(s):**

Natural log-transformed urinary protein-creatinine ratio (UPCR) ascertained at annual Chronic Renal Insufficiency Cohort research study visits.

**Analytical Approach:**

Mixed-effects regression.

**Results:**

Among 3,197 participants, the mean age was 65 years, 44% were women, and 42% self-identified as non-Hispanic Black. The median baseline estimated glomerular filtration rate was 52 mL/min/1.73 m^2^ and UPCR was 0.14 g/g. 560 patients experienced 861 episodes of AKI over a median period of 6 years, with most being stage 1 (68%). In the multivariable model that adjusted for blood pressure and renin-angiotensin system inhibitor use, each AKI episode was associated with a 3% increase in UPCR (relative change ratio, 1.03; 95% CI, 1.01-1.05; *P* < 0.01).

**Limitations:**

Hospitalized AKI with baseline CKD only, predominance of stage 1 AKI, timing of proteinuria collection was limited to annual visits, residual confounding.

**Conclusions:**

Among CKD patients, AKI was independently associated with worsening proteinuria (independent of changes in blood pressure or renin-angiotensin system inhibitor use) and may reflect residual structural damage to the kidneys. However, the increase in proteinuria among patients with CKD after AKI was small.

## Plain-Language Summary

The amount of protein in the urine is a key clinical metric used in daily practice to assess kidney health. Currently, the risk of chronic kidney disease progression is defined by the crosstabulation of glomerular filtration rate and protein in the urine. We studied 3,197 patients with chronic kidney disease and found that acute kidney injury events in the hospital were associated with an increase in urine protein levels, which may reflect residual kidney damage. Although the degree of worsening of urine protein levels was small (∼3%), these findings may guide future studies on the testing and management of urine protein after acute kidney injury.

Acute kidney injury (AKI) is common in hospitalized patients and is linked to more rapid subsequent kidney function loss.^1^^,^^2^ A proposed mechanism through which AKI contributes to the progression of chronic kidney disease (CKD) is the development of proteinuria, as identified in murine ischemia-reperfusion AKI models.^3^^,^^4^ Proteinuria may worsen after AKI because of changes in glomerular capillary permeability and the decreased reabsorption of filtered proteins by injured proximal tubules.^5^ Proteinuria may in turn directly contribute to subsequent kidney disease progression by triggering a proinflammatory response, leading to proximal tubule cell apoptosis, tubulointerstitial inflammation, and fibrosis.^6^^,^^7^

However, few studies have rigorously quantified the degree to which AKI worsens proteinuria. Parr et al^8^ reported that proteinuria increased after AKI compared with matched non-AKI controls among US veterans, but those results were based on semiquantitative dipstick measurements obtained as part of clinical care. Hsu et al^9^ quantified the change in proteinuria after AKI in a prospective study in which most participants were hospitalized shortly before enrollment. Although non-AKI study participants were included, the analysis did not fully account for potential worsening of proteinuria over time as part of the natural history of disease, even in the absence of AKI. Furthermore, AKI was captured only as present or absent, and the number of AKI episodes between annual study visits was ignored.^9^ Neither study focused exclusively on patients with CKD, who are particularly vulnerable to AKI.^10^

Using data from the multicenter, prospective Chronic Renal Insufficiency Cohort (CRIC) study,^11^^,^^12^ we aimed to quantify the independent association of AKI with the subsequent development of proteinuria among patients with CKD. This study enhances the current literature by analyzing longitudinal data collected as part of a research protocol using a mixed-effects model that accounted for changes in proteinuria over time in those who did not experience AKI, as well as factors that could influence the level of proteinuria independent of AKI, such as concurrent blood pressure measurements and renin-angiotensin system inhibitor (RASi) use. In addition, we tracked each episode of AKI rather than collapsing the study exposure into a dichotomous yes or no variable between CRIC study visits.

## Methods

### Study Population

CRIC is a prospective cohort that recruited adults with CKD from 7 clinical centers and 13 recruitment sites in the United States.^11^^,^^12^ The full study design, inclusion/exclusion criteria, and participant characteristics have previously been published.^11^^,^^13^ CRIC participants were contacted every 6 months through telephone and attended annual in-person research study visits during which blood samples were obtained for serum creatinine (Scr) evaluation and urine samples were obtained for proteinuria evaluation. Vital signs, medication use, and interim medical events were updated.

For this analysis, we included CRIC study participants from all clinical centers after July 1, 2013, when a study-wide collection of inpatient Scr values was implemented. Those who were alive, had not withdrawn from the study, and did not develop kidney failure before this date were included in this study. Study participants were followed from their first proteinuria evaluation after July 1, 2013, until the onset of kidney failure (defined as the date of maintenance dialysis initiation or receipt of kidney transplant), death from any cause, or date of administrative censoring (December 1, 2021).^14^

Institutional review boards at the participating institutions approved the study protocol and informed consent was obtained from all study participants.

### Exposure

The primary exposure was the number of AKI events since the last annual CRIC visit. AKI events were captured during hospitalizations. Hospitalizations among CRIC study participants were ascertained at annual study site visits and interim 6-month phone calls along with active study personnel review of hospital records. Scr values measured during these hospitalizations were then systematically abstracted to determine AKI status. No Scr values obtained from annual CRIC visits were used to define AKI. Adapted from the KDIGO (Kidney Disease: Improving Global Outcomes) AKI guidelines, AKI was defined as a 50% or greater increase in inpatient Scr measurement from nadir to peak.^15^ Scr values from annual CRIC visits were not used for AKI case detection to avoid misclassification of CKD progression as AKI events. Similar to other AKI studies using CRIC data, the absolute change in Scr by ≥0.3 mg/dL was not applied to define AKI.^16^^,^^17^ This more stringent definition of AKI was used as the cohort had baseline CKD, and patients with higher baseline levels of Scr are more likely to have small absolute changes in Scr values as a result of inherent laboratory and biological variability, leading to misclassification of false-positive AKI diagnoses.^18^

### Outcome

The primary outcome of interest was proteinuria defined by urinary protein- creatinine ratio (UPCR) in grams per gram (natural log-transformed in the analysis to account for skewed distribution). Proteinuria was assessed using 24-hour urine collections or random spot urine samples collected at annual in-person CRIC study visits. Urine protein was quantified using a colorimetric reaction on the UniCel DxC-800 analyzer (Beckman Coulter) and urine creatinine was measured using a Jaffe reaction on the UniCel DxC-800 analyzer (Beckman Coulter).

### Covariates

The following covariates were selected a priori based on clinical and pathophysiologic considerations. Age at baseline, self-identified sex and race/ethnicity (non-Hispanic Black, non-Hispanic White, Hispanic, and other), and CRIC clinical center were not time updated. Estimated glomerular filtration rate (eGFR), systolic blood pressure (SBP), diabetes status, number of blood pressure medications, and RASi use were incorporated as time-updated variables. Diabetes status was defined as a fasting glucose level of 126 mg/dL or higher, a random glucose level of 200 mg/dL or higher, or self-reported use of insulin or oral diabetic medication. eGFR was assessed using the 2021 Scr-based CKD-EPI (Chronic Kidney Disease Epidemiology Collaboration) equation.^19^ Blood pressure measurements were obtained at annual study visits per a standardized protocol.^20^ Both angiotensin-converting enzyme inhibitors and angiotensin receptor blockers were considered RASi.

### Analysis

We summarized characteristics of study participants who had at least 1 episode of AKI during follow-up and compared them with that of those who never experienced AKI during follow-up.

Our first analysis was a descriptive one limited to CRIC study participants who had AKI. We examined the first episode of AKI for these study participants and presented raw data for key variables such as UPCR and SBP before and after the first AKI episode. Comparisons for this descriptive analysis were made using the paired *t* test and Wilcoxon signed rank test based on data distribution.

Our main analysis relied on mixed-effects regression and examined the change in natural log-transformed UPCR. This approach was chosen to account for within-person variations in proteinuria by specifying a random intercept and random slope of time (change in age) for an individual and defining the average change in UPCR based on every AKI episode for a given study participant.

In the unadjusted model, the following equation was applied:

Yit=NaturalloguPCRratioforpersoniattimet

$$=\beta_{0}+\beta_{0i}+\beta_{1}{\text{AKI}}_{it}+\beta_{2}\left({\text{AGE}}_{it}-{\text{AGE}}_{i,baseline}\right)+\beta_{2i}\left({\text{AGE}}_{it}-{\text{AGE}}_{i,baseline}\right)+\varepsilon it\text{,}$$

where *β*_*0*_ is the mean natural log UPCR at baseline for the entire cohort assuming no AKI. *β*_*0i*_ is a random effect for the difference in mean natural log UPCR for person *i* from the cohort. AKI_*it*_ is the number of AKI episodes experienced by person *i* between time *t − 1* and *t* (not just whether AKI occurred or not). AGE_*i,baseline*_ indicates the age at study baseline, so AGE_*it*_ - AGE_*i,baseline*_ denotes the age of each person *i* at time *t* compared with their age at baseline and accounts for the time in study. β_2_ accounts for the natural progression of proteinuria over time. ε_*it*_ is a random error term for person *i* at time *t.*

In the fully adjusted model, we used the following equation:

Yit=NaturalloguPCRratioforpersoniattimet

$$=\beta_{0}+\beta_{0i}+\beta_{1}{\text{AKI}}_{it}+\beta_{2}\left({\text{AGE}}_{it}-{\text{AGE}}_{i,baseline}\right)+\beta_{2i}\left({\text{AGE}}_{it}-{\text{AGE}}_{i,baseline}\right)+\beta_{3}{\text{AGE}}_{i,baseline}+\beta_{4}{\text{RACE}}_{i}+\beta_{5}{SEX}_{i}+\beta_{6}{\text{CCID}}_{i}+\beta_{7}{\text{eGFR}}_{it}+\beta_{8}{\text{DIABETES}}_{it}+\beta_{9}{\text{Antihypertensives}}_{it}+\beta_{10}{\text{SBP}}_{it}+\beta_{11}{\text{RASi}\;\text{use}}_{it}+\varepsilon it$$

RACE*i*, SEX*i,* and CCID*i* indicate race/ethnicity (based on category), sex, and CRIC clinical site identification number (based on category) of person *i*. eGFR_*it*_, DIABETES_*it,*_ Antihypertensives_*it*_, SBP_*it*_, and RASi use_*it*_ denote time-updated variables of eGFR, presence of diabetes, number of antihypertensive medications, SBP, and use of RASi for person *i* at time *t*, respectively.

To evaluate the impact of potential proteinuria management strategies on this relationship, we further assessed the association between AKI and change in UPCR after excluding RASi use and SBP and restricting the cohort to those without RASi use at baseline.

In the secondary analysis, we explored the association of AKI severity and duration with changes in proteinuria. KDIGO creatinine-based definitions were applied to define AKI severity, in which those with stage 1 AKI had 1.5- to 1.9-fold increase in Scr, stage 2 AKI had 2- to 2.9-fold increase in Scr, and stage 3 AKI had 3-fold or higher increase in Scr, Scr ≥ 4 mg/dL, or initiation of kidney replacement therapy.^15^ AKI duration was dichotomized by <2 versus ≥2 days and <3 versus ≥3 days when inpatient Scr was 50% or greater than the nadir (ie, recovery from AKI was defined as when Scr was no longer 50% or greater than the nadir).

All analyses were conducted using SAS software, version 9 (SAS Institute). Missingness was noted in <5% of the variables, and complete case analysis was applied.

## Results

The final cohort included 3,197 participants with a median (interquartile range) follow-up time of 6 (3-7) years. Baseline characteristics of the cohort overall and based on AKI status are in Table 1. Mean age was 65 years, 44% were women, and 42% self-identified as non-Hispanic Black. The median baseline eGFR was 52 mL/min/1.73 m^2^ and UPCR was 0.14 g/g. The prevalence of diabetes was 54%, RASi use was reported in 66%, and the median number of blood pressure medications was 2 at the baseline visit. The average baseline SBP was 127 mm Hg.

**Table 1:** Baseline Characteristics of Those Who Experienced AKI During Follow-up Versus Those Who Did Not Experience AKI During Follow-up

Characteristics	Overall N = 3,197	No AKI N = 2,637	AKI N = 560
Age (in y)	65 ± 10	65 ± 10	66 ± 9
Female	1402 (44%)	1134 (43%)	268 (48%)
Race/ethnicity			
Non-Hispanic Black	1341 (42%)	1075 (41%)	266 (48%)
Non-Hispanic White	1418 (44%)	1189 (45%)	229 (41%)
Hispanic	335 (11%)	281 (11%)	54 (10%)
Other	103 (3%)	92 (4%)	11 (2%)
Baseline eGFR (mL/min/1.73 m^2^)	52 (41-63)	52 (41-63)	51 (40-60)
Baseline urinary protein-creatinine ratio (g/g)	0.14 (0.06-0.50)	0.13 (0.06-0.49)	0.16 (0.07-0.57)
Diabetes status at baseline	1712 (54%)	1340 (51%)	372 (66%)
No. of blood pressure medications at baseline	2 (1-3)	2 (1-3)	3 (2-4)
RASi use at baseline	2077 (66%)	1680 (64%)	397 (71%)
Systolic blood pressure (mm Hg) at baseline	127 ± 20	126 ± 19	128 ± 20
Follow-up time in y	6 (3-7)	6 (3-7)	6 (4-7)

*Note*: Data are presented as n (%) if categorical, mean ± standard deviation if continuous, and median (lower interquartile range-upper interquartile range) if skewed.

Abbreviations: AKI, acute kidney injury; eGFR, estimated glomerular filtration rate; RASi, renin-angiotensin system inhibitor including angiotensin-converting enzyme inhibitor and angiotensin receptor blocker.

Among the 3,197 participants, 560 patients experienced AKI at some point during follow-up, whereas 2,637 did not. A total of 861 AKI episodes were tallied. One hundred one (18%) participants were identified as having 2 AKI episodes, and 61 (11%) participants had 3 or more AKI episodes. The severity of these 861 AKI episodes was predominantly stage 1 (n = 586; 68%) rather than stage 2 (n = 223; 26%) or stage 3 (n = 52; 6%). According to the Clinical Classifications Software, a tool developed by the Agency for Healthcare Research and Quality, for grouping patient diagnoses into a manageable number of clinically meaningful categories and CRIC adjudication, the distribution of reasons for hospitalization for the AKI cases is as follows: 20% cardiovascular related, 16% infection related, and 11% related to heart failure.

Study participants who developed AKI were more likely to be women, non-Hispanic Black, and have diabetes. At baseline, they also had a higher number of antihypertensive medications, more RASi use, higher UPCR, and higher SBP. During follow-up, study participants had a median of 5 UPCR measurements. The median number of days between AKI and the previous UPCR measurement was 218 and the median number of days between AKI and the subsequent UPCR measurement was 175.

Results from our first descriptive analysis are shown in Table 2. Among the CRIC study participants who did develop AKI, after the first episode of AKI, the median UPCR increased from 0.24-0.29 g/g, and RASi use decreased from 72%-53%.

**Table 2:** Change in Proteinuria and Change in Management Strategies Before and After First AKI (n = 560)

Proteinuria and Management Strategies	Closest CRIC Visit Before AKI	Closest CRIC Visit After AKI	*P* Value
Median urinary protein-creatinine ratio (g/g)	0.24 (0.11-0.79)	0.29 (0.11-0.97)	<0.01
Mean systolic blood pressure (mm Hg)	129 ± 21	129 ± 22	0.91
Median no. of antihypertensives	3 (2-4)	3 (2-4)	0.02
Prevalence of RASi use	72%	53%	<0.01

*Note*: Data are presented as n (%) if categorical, mean ± standard deviation if continuous, and median (lower interquartile range-upper interquartile range), if skewed.

Abbreviations: AKI, acute kidney injury; CRIC, Chronic Renal Insufficiency Cohort; RASi, renin-angiotensin system inhibitor.

Results from our main analysis are shown in Table 3. The univariable mixed-effects model showed that each episode of AKI was associated with a 3% increase in UPCR (relative change ratio, 1.03; 95% confidence interval [CI], 1.02-1.05; *P* < 0.01). The full multivariable mixed-effects model showed that after adjusting for potential confounding factors, the association of AKI with UPCR was similar and remained statistically significant (relative change ratio, 1.03; 95% CI, 1.01-1.05; *P* < 0.01). No substantial changes in the strength of the association between AKI and proteinuria were observed after excluding RASi use and SBP from our multivariable mixed models (ie, similar relative estimates, 95% CIs, and *P* values) or by limiting the analysis to those without baseline RASi use (Table S1).

**Table 3:** Mixed-Effects Models of Change in Log-Transformed UPCR Per AKI Episode (Univariable, Multivariable, Multivariable Excluding RASi Use, and Multivariable Excluding SBP) With No AKI As Reference

No AKI (Reference)	Relative Change Ratio^a^ (95% CI)	*P* Value
Univariable model per AKI episode (n = 3,197)	1.03 (1.02-1.05)	<0.01
Multivariable model^b^ (n = 3,173)	1.03 (1.01-1.05)	<0.01
Multivariable model excluding RASi use (n = 3,173)	1.03 (1.02-1.05)	<0.01
Multivariable model excluding SBP (n = 3,180)	1.03 (1.01-1.04)	<0.01

Abbreviations: AKI, acute kidney injury; CI, confidence interval; eGFR, estimated glomerular filtration rate; RASi, renin-angiotensin system inhibitor; SBP, systolic blood pressure; UPCR, urinary protein-creatinine ratio.

a The relative change ratio is the exponentiated regression coefficient, representing the relative mean change in UPCR (ie, a ratio of 2 means) for every AKI episode compared with no AKI. Subtracting 1 from the relative change ratio and multiplying by 100 represents the percent change in mean UPCR.

b Multivariable model includes age, sex, race/ethnicity, clinical center identification number, eGFR, hypertension, diabetes, RASi use, number of blood pressure drugs, and systolic blood pressure.

The results of our secondary analysis are shown in Table 4. The full multivariable models showed similar changes in UPCR based on AKI stage (relative change ratio, 1.03). A longer AKI duration was associated with higher relative change ratios in proteinuria (1.02 [95% CI, 0.99-1.03] for AKI < 2 days, 1.04 [95% CI, 1.02-1.07] for AKI ≥ 2 days, and 1.07 [95% CI, 1.04-1.11] for AKI ≥ 3 days) compared with no AKI.

**Table 4:** Mixed-Effects Models of Change in Log-Transformed UPCR Per AKI Episode Based on AKI Stage and Duration With No AKI As Reference

Per AKI Episode With No AKI As Reference	Univariable Model (n = 3,197)		Multivariable Model^a^ (n = 3,173)		Multivariable Model Excluding RASi Use (n = 3,173)		Multivariable Model Excluding Systolic Blood Pressure (n = 3,180)	
	Relative Change Ratio^b^ (95% CI)	*P* Value	Relative Change Ratio^b^ (95% CI)	*P* Value	Relative Change Ratio^b^ (95% CI)	*P* Value	Relative Change Ratio^b^ (95% CI)	*P* Value
Based on stage								
Stage 1	1.03 (1.01-1.05)	<0.01	1.03 (1.01-1.05)	<0.01	1.03 (1.01-1.05)	<0.01	1.02 (1.00-1.04)	0.03
Stage 2-3	1.04 (1.01-1.07)	0.02	1.03 (1.00-1.06)	0.06	1.03 (1.00-1.07)	0.03	1.03 (1.00-1.06)	0.05
Based on duration								
AKI < 2 d	1.02 (0.99-1.04)	0.19	1.02 (0.99-1.03)	0.13	1.02 (1.00-1.04)	0.11	1.01 (0.98-1.03)	0.46
AKI ≥ 2 d	1.05 (1.02-1.07)	<0.01	1.04 (1.02-1.07)	<0.01	1.05 (1.02-1.07)	<0.01	1.04 (1.02-1.07)	<0.01
AKI < 3 d	1.01 (0.99-1.03)	0.19	1.01 (1.00-1.03)	0.13	1.02 (1.00-1.04)	0.09	1.01 (0.99-1.03)	0.45
AKI ≥ 3 d	1.08 (1.05-1.12)	<0.01	1.07 (1.04-1.)	<0.01	1.08 (1.04-1.11)	<0.01	1.08 (1.04-1.11)	<0.01

Abbreviations: AKI, acute kidney injury; CI, confidence interval; eGFR, estimated glomerular filtration rate; RASi, renin-angiotensin system inhibitors; UPCR, urinary protein-creatinine ratio.

a Multivariable model includes age, sex, race/ethnicity, clinical center identification number, eGFR, hypertension, diabetes, RASi use, number of blood pressure drugs, and systolic blood pressure.

b The relative change ratio is the exponentiated regression coefficient, representing the relative mean change in UPCR (ie, a ratio of 2 means) for every AKI episode compared with no AKI. Subtracting 1 from the relative change ratio and multiplying by 100 represents the percent change in mean UPCR.

## Discussion

Using data from CRIC, our study indicated that AKI is an independent risk factor for subsequent proteinuria in patients with CKD even after accounting for the natural progression of proteinuria over time, changes in RASi use, as well as changes in SBP. In addition to the direction of association, the magnitude of proteinuria is noteworthy; each AKI event was associated with a 3% increase in proteinuria (with the upper limit of 95% CI at 5%).

Our results are consistent with prior findings noting the association of AKI with new-onset or worsening proteinuria.^8^^,^^9^^,^^21^, ^22^, ^23^, ^24^, ^25^, ^26^, ^27^, ^28^ In a retrospective case series of 51 patients with posttraumatic AKI requiring kidney replacement therapy, 25% were reported to have proteinuria.^23^ Extended follow-up of participants in the Randomized Evaluation of Normal versus Augmented Levels of renal replacement therapy trial showed the prevalence of albuminuria in ∼40% of survivors.^24^ Using data from the AKI Risk in Derby case-control study, Horne et al^25^, ^26^, ^27^, ^28^ reported increased proteinuria among those with AKI. However, these studies lacked systemic assessment of proteinuria before AKI.^23^, ^24^, ^25^, ^26^, ^27^, ^28^ In a cohort of 90,614 veterans, Parr et al^8^ found higher odds of 1+ or a greater dipstick measurement during follow-up (odds ratio, 1.20-1.39) among those with AKI compared with a matched population without AKI. Bonde et al^21^ evaluated 354 patients with AKI and no baseline proteinuria and found that ∼60% of patients developed de novo proteinuria within 1 year after hospital discharge. Su et al^22^ found that 33.9% of patients without proteinuria at baseline developed proteinuria after AKI and 17.4% of patients with baseline proteinuria had worsening proteinuria among 6,206 Chinese patients. However, proteinuria evaluation in these studies occurred as a part of routine care, which only occurred in ∼20% of the population.^21^^,^^22^ Selection bias could potentially lead to an exaggerated observed effect of worsening proteinuria after AKI, as evaluation likely occurs in more severe disease. In addition, the method of proteinuria evaluation was often only semiquantitative urine dipstick evaluations rather than UPCR quantification.^8^^,^^21^^,^^22^ Of these studies, only Parr et al^8^ and Horne et al^25^, ^26^, ^27^, ^28^ had control patients without AKI to be able to account for proteinuria that results from natural CKD progression and critical illness.

Most existing studies also did not consistently and rigorously account for changes in proteinuria management strategies after AKI, such as changes in RASi use and blood pressure control. Parr et al^8^ evaluated for changes in postdischarge blood pressure in AKI versus no AKI group but not changes in RASi. Su et al^22^ captured changes in RASi use after AKI but did not account for changes in blood pressure. Proteinuria management strategies are recommended for patients at risk for kidney disease progression but often interrupted after AKI owing to concerns about hyperkalemia during periods of decreased kidney function and risk of delayed recovery from AKI or development of recurrent AKI. Concurrent assessment of SBP, RASi use, and proteinuria is key to understanding the degree to which any increase in proteinuria observed after AKI could be ascribed to these management choices. To isolate the potential contribution of SBP control and RASi use to change in proteinuria, we analyzed the multivariable model and then removed these variables to estimate their impact as well as restricted the analysis to those without RASi use at baseline.

Only one other prior study by Hsu et al^9^ quantified the change in proteinuria after AKI while accounting for proteinuria in non-AKI controls and proteinuria management strategies. Most of the study participants in that study were hospitalized before the start of the prospective observation period. Compared with our study population, the median baseline eGFR was higher (63 compared with 52 mL/min/1.73 m^2^) and the UPCR was lower (0.12 g/g compared with 0.14 g/g). There were more AKI stage 2-3 patients (50% compared with 32%). They found that an episode of AKI was associated with a 9% increase in UPCR (relative change ratio, 1.09; 95% CI, 1.02-1.06).

Both Hsu et al^9^ and this current study used data collected as part of a research protocol, which reduces the risk of ascertainment bias compared with studies that rely on data collected as part of routine clinical care.^8^^,^^21^, ^22^, ^23^, ^24^ SBP and RASi use were tracked at the same time points as when proteinuria was quantified, which is valuable because physicians may reduce the use of RASi or liberalize blood pressure control after AKI out of concern for recurrent AKI, and that may be an alternate explanation for any observed increase in proteinuria level after AKI. This study also benefited from the extended follow-up time of 6 years, which facilitated additional cases of AKI identification, improving the study power.^16^

Our study advances the literature in several ways. We defined AKI episodes as a continuous variable rather than the presence or absence of AKI between study visits and thus more accurately quantified changes per AKI episode. In addition, we incorporated an additional term in the mixed-effects models that accounts for the time in study and by extension, the natural progression of proteinuria in CKD. Based on an exclusively CKD cohort, these results are particularly relevant to patients at higher risk of adverse events from AKI compared with the non-CKD general population.

Limitations of our analysis include the fact that UPCR measurements were only obtained at annual CRIC study visits, so we were unable to study at a more granular level how proteinuria changes during and soon after AKI. Furthermore, we accounted for changes in RASi use but not changes in RASi dosing. AKI was defined using Scr values collected during hospitalizations only, so outpatient AKI cases were missed. In addition, if the nadir hospital Cr was higher than the usual outpatient baseline Cr, then these AKI cases may have also been missed. Results may not be generalized to those without CKD or higher AKI severity, as CRIC only enrolled patients with CKD, and the cohort had predominantly episodes of AKI stage 1. The strength of the association may be underestimated, especially given that proteinuria is associated with a high degree of random measurement error.

Although observational studies cannot demonstrate causality, the totality of evidence is consistent with the hypothesis that the increased proteinuria observed after AKI represents residual structural damage to the kidneys even after accounting for the natural progression of proteinuria over time, changes in RASi use, as well as changes in SBP. These results are consistent with pathophysiology studies that indicate that although considerable intrinsic repair capacity exists after AKI, a subpopulation of cells may fail to undergo normal repair and ultimately manifest as residual proteinuria.^29^ Our secondary results of the stronger effect seen among AKI of a longer duration may be associated with more residual renal parenchymal damage than AKI of a shorter duration, which may be more enriched with patients with clinical prerenal azotemia.

Our findings contribute to the literature by rigorously analyzing the association between an episode of AKI and proteinuria among patients with CKD in a prospective cohort study to minimize ascertainment bias. Our data also highlight that the increase in proteinuria was small. Patients found to have proteinuria after AKI may benefit from being managed similar to those with known proteinuric CKD.^30^, ^31^, ^32^ Regardless of the exact pathophysiology, our results support current practice guidelines that recommend assessing proteinuria after AKI to improve risk stratification, which would help guide the management of survivors.^33^ The field should move beyond epidemiologic studies of associations to interventional studies to evaluate whether treatment of proteinuria can improve outcomes after AKI.
