# Supplementary material for: Quantifying Change in Proteinuria After Acute Kidney Injury Among Patients With Chronic Kidney Disease: Findings From the Chronic Renal Insufficiency Cohort (CRIC) Study
Source: Kidney Med. 2026 Jan 29;8(4):101272. doi: 10.1016/j.xkme.2026.101272 (PMC13019561; doi:10.1016/j.xkme.2026.101272)
Supplement: Supplementary File (PDF) — Table S1. [file mmc1.docx]

Supplemental Table 1: Mixed effects models of change in log-transformed urine protein to creatinine ratio per AKI episode with no AKI as reference restricted to patients without RASi use at baseline

| No AKI (ref) | Relative Change Ratio^†^  (95% CI) | p-value |
| --- | --- | --- |
| Univariable model per AKI episode (n=1,095) | 1.04 (1.02, 1.07) | <0.01 |
| Multivariable model* (n=1,089) | 1.04 (1.02, 1.07) | <0.01 |

Abbreviations: AKI: acute kidney injury; CI: confidence interval, RASi: renin angiotensin system inhibitors

*****Multivariable model includes age, sex, race/ethnicity, clinical center identification number, eGFR, hypertension, diabetes, RASi use, number of blood pressure drugs, and systolic blood pressure

†The relative change ratio is the exponentiated regression coefficient, representing the relative mean change in uPCR (i.e., a ratio of two means) for every AKI episode comparing with no AKI. Subtracting 1 from the relative change ratio and multiplying by 100 represents the percent change in mean uPCR.
